# The role of macrophages in hypertrophic scarring: molecular to therapeutic insights

**DOI:** 10.3389/fimmu.2025.1503985

**Published:** 2025-03-28

**Authors:** Lele Shen, Yao Zhou, Jie Gong, Hongqiao Fan, Lifang Liu

**Affiliations:** Department of Galactophore, The First Hospital of Hunan University of Chinese Medicine, Changsha, Hunan, China

**Keywords:** hypertrophic scar, macrophages, inflammation, signaling molecules, M1, M2

## Abstract

Hypertrophic Scar (HS) is a common fibrotic disease of the skin, usually caused by injury to the deep dermis due to trauma, burns, or surgical injury. The main feature of HS is the thickening and hardening of the skin, often accompanied by itching and pain, which seriously affects the patient’s quality of life. Macrophages are involved in all stages of HS genesis through phenotypic changes. M1-type macrophages primarily function in the early inflammatory phase by secreting pro-inflammatory factors, while M2-type macrophages actively contribute to tissue repair and fibrosis. Despite advances in understanding HS pathogenesis, the precise mechanisms linking macrophage phenotypic changes to fibrosis remain incompletely elucidated. This review addresses these gaps by discussing the pathological mechanisms of HS formation, the phenotypic changes of macrophages at different stages of HS formation, and the pathways through which macrophages influence HS progression. Furthermore, emerging technologies for HS treatment and novel therapeutic strategies targeting macrophages are highlighted, offering potential avenues for improved prevention and treatment of HS.

## 1 Introduction

Hypertrophic scar (HS) is a fibrotic disease of the skin, usually caused by abnormal tissue repair after burns, trauma, or surgery (1). The clinical manifestations of HS are bright red skin surface, protruding from the surrounding normal skin tissues, and localized thickening. Itching, localized numbness, and sensory abnormalities produced by HS have a severe impact on the quality of life and mental health of patients (1, 2). HS formation is a complex and challenging clinical problem that affects about 100 million patients in developed countries alone. The incidence of post-burn HS has been reported to range from 32% to 72% (3, 4). Although many studies have been devoted to exploring the mechanisms of HS, its exact pathophysiologic processes are still not fully characterized.

The formation of HS is a complex and multistage process that usually includes hemostasis, inflammation, proliferation, and remodeling phases. Macrophages are an essential component of the innate immune system. They play a crucial role in tissue repair and scar formation. Macrophages exhibit different phenotypes and functions at different stages of HS formation. M1 macrophages, also known as classically activated macrophages, primarily mediate pro-inflammatory and antimicrobial responses, producing cytokines such as TNF-α, IL-6, and IL-1β, which amplify inflammation and recruit additional immune cells (5, 6). As the inflammatory response subsides, macrophages transition to the M2 phenotype, which promotes tissue repair, extracellular matrix remodeling, and fibrosis through the secretion of anti-inflammatory cytokines and growth factors (7, 8). However, macrophage-targeted therapies for HS face significant challenges, including the complex plasticity of macrophage phenotypes, their dynamic changes during the different stages of HS formation, and the need for specific delivery systems to target macrophages without affecting surrounding tissues. Precision therapy targeting macrophages has made significant progress in tumors and rheumatic diseases (9, 10). However, the exact mechanism of macrophage action in HS remains under-revealed.

In this review, we summarize the pathological process of HS formation and the phenotypic changes that occur in macrophages at various stages of HS formation. In addition, we explore macrophage-influenced pathways in HS. Finally, we summarize the therapeutic strategies for HS, including emerging technologies and macrophage-targeted treatment approaches, and discuss the specific challenges associated with these strategies. This review systematically integrates research findings spanning from molecular mechanisms to therapeutic strategies, based on comprehensive searches of databases such as PubMed and Web of Science, to identify critical gaps and highlight potential advancements in the treatment of HS. We hope that it will help develop drugs of potential treatment value for HS and provide theoretical support for developing more effective therapeutic strategies.

## 2 Methods

This review is based on a systematic literature search conducted in the PubMed and Web of Science databases using keywords such as “hypertrophic scar,” “macrophages,” “fibrosis,” and “therapeutic strategies,” combined with Boolean operators (AND/OR). The search was performed on August 24, 2024. The inclusion criteria were as follows: (1) studies focusing on the role of macrophages in the mechanisms of HS and therapeutic strategies for HS; (2) original research, including *in vivo*, *in vitro*, and clinical studies; and (3) studies published in English to ensure accessibility and practical usability. The exclusion criteria were as follows: (1) studies unrelated to HS; (2) conference abstracts, pathological reports, or review articles; and (3) articles with insufficient data quality or poor study design. The screening process consisted of two steps: title and abstract screening followed by full-text evaluation. These steps were implemented to ensure the relevance, reliability, and quality of the included studies.

## 3 Pathological mechanisms of HS formation

Routine wound healing consists of four phases: hemostasis, inflammation, proliferation, and remodeling, each of which overlaps and differs in time and space (1).The formation of HS is the result of an abnormally active and dysregulated event during the wound healing phase, leading to an abnormal accumulation of extracellular matrix (ECM). Next, we will explore the characteristics of HS at different stages of formation.

### 3.1 Hemostasis

Hemostasis is the first step in wound repair (11). Damaged endothelial cells release substances such as endothelin to constrict vascular smooth muscle and reduce bleeding (12, 13). Generally, forming a blood clot involves primary and secondary hemostatic processes. During initial hemostasis, platelet receptors interact with ECM proteins, such as fibronectin and collagen, to form platelet plugs that promote adhesion to the vessel wall (14). Secondary hemostasis depends on a coagulation factor cascade reaction that activates thrombin (15). Thrombin induces the conversion of fibrinogen to fibrin, which results in the formation of a stable fibrin network. This fibrin network and fibronectin, vitronectin, and thrombospondin form an insoluble clot (16).

**Figure 1:**
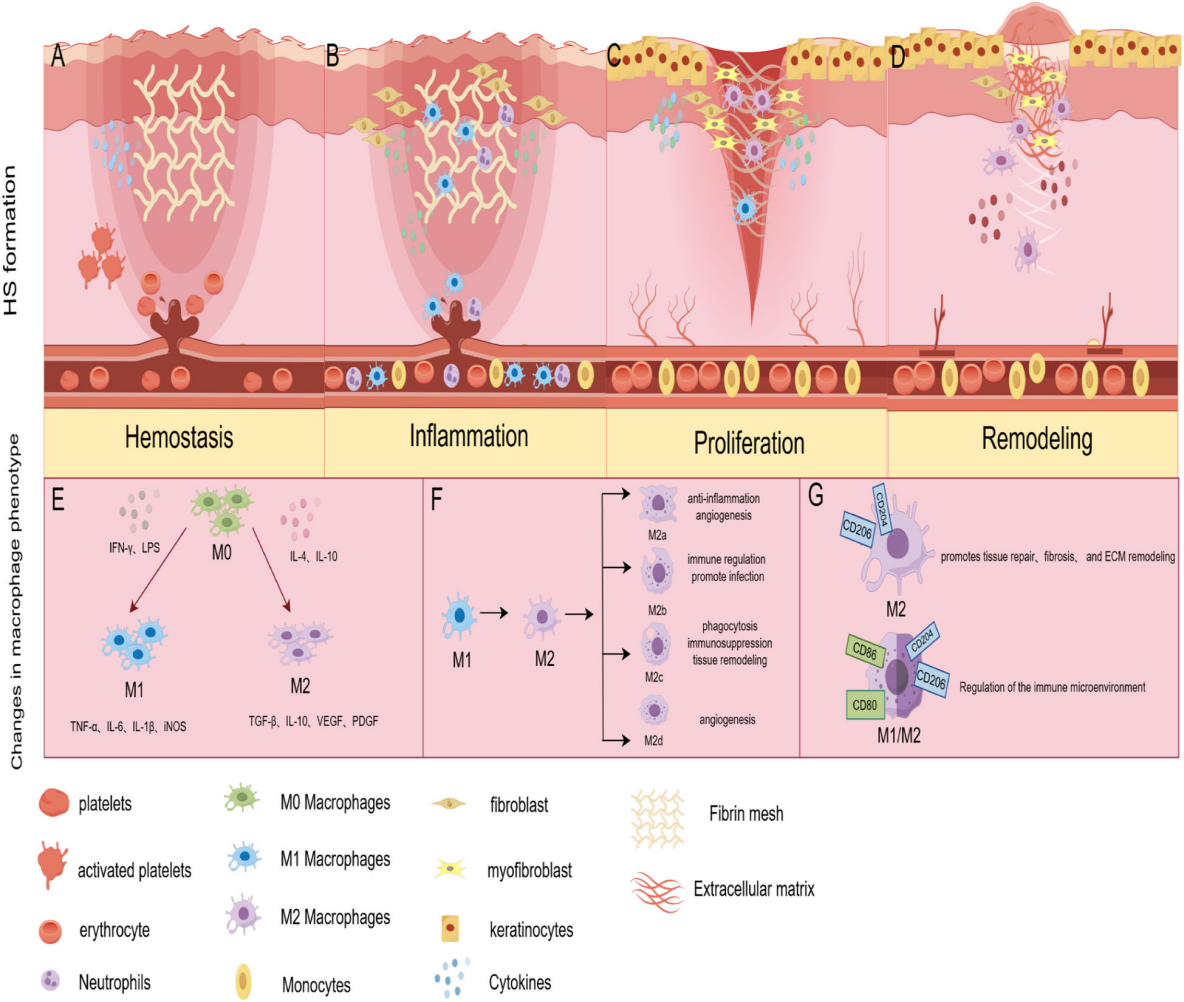
Pathological mechanisms of HS formation and phenotypic changes in macrophages during the HS formation phase. **(A)** Hemostasis: aberrant release of pro-fibrotic molecules leads to a sustained inflammatory response and excessive fibrin network formation. **(B)** Inflammation: high levels of inflammatory infiltrate leads to fibroblast activation. **(C)** Proliferation: overactive keratinocytes, dermal fibroblasts, and macrophages promote ECM deposition and excessive angiogenesis. **(D)** Remodeling: fibroblasts remodel the deposited ECM, myofibroblasts cause overall wound contraction; MMPs regulate fibroblast proliferation and are involved in ECM degradation. fibroblasts cause overall wound contraction; MMPs regulate fibroblast proliferation and participate in ECM degradation. **(E)** During HS formation, M0 macrophages respond to an initial stimulus to polarize to M1 and M2 phenotypes, with the M1 phenotype playing a significant role in the early stages. **(F)** Macrophages undergo an M1 to M2 phenotypic transition, or conversion of the M2 subtype, in the intermediate stages of HS formation; **(G)** Macrophages are dominated by the M2 phenotype in the later stages of injury and may develop a mixed M1/M2 phenotype. (By Figdraw).

During hemostasis, platelet activation and release of growth factors, the intensity and duration of the inflammatory response, and deposited fibrin are closely related to the formation of HS (17). The initial hemostatic event occurs when platelets produce pro-fibrotic growth factors such as PDGF, VEGF, TGF-β1, and CTGF. Moderate release of these factors promotes hemostasis (11). Overproduction of pro-fibrotic factors can lead to excessive cell proliferation and fibrous tissue production. Previous studies have found that the use of platelet-rich plasma (PRP) for treating various types of scars is increasing. PRP attenuates the fibrotic process by decreasing the levels of pro-fibrotic markers Transforming Growth Factor Beta 1 (TGF-β1), smooth muscle actin α (α-SMA), Collagen Type I (COL-I), and Matrix Metalloproteinase-9 (MMP-9) (18, 19). In addition, these pro-fibrotic molecules may induce excessive inflammation. This may lead to the overproduction of ECM, abnormal fibroblast differentiation, and inappropriate matrix remodeling, which may promote the formation of HS (1). Overall, the release of abnormal pro-fibrotic molecules during the hemostatic phase, and the sustained inflammatory response lead to excessive fibrin network formation in HS (**Figure 1A**), which ultimately promotes HS (20).

### 3.2 Inflammation

HS is formed due to skin damage in the reticular dermis (21). The inflammatory response in the reticular dermis begins immediately at the time of injury and varies in duration depending on the degree of injury (12). The study suggests excessive inflammation is the pathologic basis for HS formation (22). Increased expression of interleukins, interferons, and growth factors released by immune cells, such as neutrophils and macrophages, can activate fibroblasts (**Figure 1B**), which are involved in the formation of HS (23, 24).

Previous studies have shown the most apparent effect of IL-6 in promoting human HS formation. IL-6 levels were significantly elevated in burned HS fibroblasts (25). IL-6 enhances VEGF expression in macrophages, keratinocytes, and fibroblasts, ultimately leading to scarring (26). In addition to IL-6, inflammatory factors such as IL-1β, IL-4, IL-17, and IL-13 were highly expressed in HS (27, 28). Interestingly, another point was made that pro-inflammatory cytokines contribute to wound healing. Inadequate pro-inflammatory responses slow the wound-healing process (29, 30). This delay in the inflammatory response during the acute phase of early healing and its role in HS formation deserves further investigation.

In contrast to IL-6, the expression of IL-10, IL-18, and IL-37 was lower in HS. IL-10 was found to regulate the TLR4/NF-κB pathway in dermal fibroblasts via the IL-10R/STAT3 axis. Through this mechanism, IL-10 attenuated the deleterious effects of LPS on wound healing, further reducing scar formation and skin fibrosis (31). This suggests that IL-10 may mediate the TLR4/NF-κB pathway to exert anti-scarring effects.IL-18 and IL-37 are members of the IL-1 family (32). The available evidence indicates that IL-18 and IL-37 have the potential as new treatments for pathologic scarring (33, 34). However, further research is required to fully ascertain their potential and elucidate these cytokines’ optimal targeting.

### 3.3 Proliferation

Overactive keratinocytes, dermal fibroblasts, vascular endothelial cells, and macrophages promote HS progression during proliferative (**Figure 1C**). The standard proliferative period of wound healing begins about three days after injury and may last 2 ~ 3 weeks. Indeed, from 12 hours after injury, keratinocytes are activated by changes in the microenvironment of hydrogen peroxide, pathogens, growth factors, and cytokines, which result in a high degree of activation, hyperproliferation, and aberrant differentiation in the HS (35). In this process, keratinocytes produce pro-fibrotic molecules such as TGF-βand PDGF (36). These molecules induce fibroblasts to respond to the pro-fibrotic environment by producing more extracellular matrix (ECM) proteins or differentiating into myofibroblasts, accelerating scar formation (11). Fibroblasts in the upper and deeper dermis have different functions (37). Where fibroblasts in the upper dermis contribute to re-epithelialization, fibroblasts in the deeper spectrum contribute to ECM deposition. In HS proliferative events, endothelial dysfunction and altered expression of angiogenic genes such as endothelin and angiopoietin can lead to excessive angiogenesis (38). Studies have shown that excessive angiogenesis can increase collagen deposition (39). Macrophages play an essential role in this process by participating in the remodeling of neovascularization, phagocytosis of excess blood vessels, and inhibition of the angiogenic response (40).

### 3.4 Remodeling

The main event during the HS remodeling phase is the dysregulation of the balance between ECM collagen synthesis and degradation (41). This is accomplished by regulating key MMPs (42). MMPs are expressed by macrophages, fibroblasts, and keratin-forming cells (43). Previous studies have found that MMP2 and MMP9, enzymes essential for remodeling the ECM, are significantly elevated in the pathological microenvironment of HS (44, 45). MMP1 and MMP7 are downregulated during HS formation (46). A study observed that fibroblast proliferation and migration can be inhibited by reducing MMP-9 expression, thereby reducing fibrotic scar formation (47). Altered expression of these MMPs results in reduced degradation of ECM components, including COL-1, COL-3, and fibronectin (48). Additionally, macrophage signaling pathways play a crucial role in regulating HS remodeling. The Notch signaling pathway controls the expression of Smad, α-SMA, and collagen in HS fibroblasts to some extent (49, 50). A study using RBP-J knockout mice demonstrated that inhibition of Notch signaling in macrophages suppresses the inflammatory response and reduces collagen deposition, leading to better wound healing and reducing fibrosis (51). The above studies suggest macrophage-derived MMPs and their intracellular signaling control tissue remodeling during skin wound repair (**Figure 1D**). However, the precise role of macrophages in scar formation remains insufficiently understood.

## 4 Phenotypic changes in macrophages during the HS formation phase

### 4.1 Initial response and early phenotype

In response to initial stimuli such as pathogens, cytokines, or injury signals, macrophages typically polarize rapidly into either a classically activated (M1-type) or alternatively activated (M2-type) phenotype (52) (**Figure 1E**). This initial response typically occurs within a few hours to a few days, representing a rapid immune reaction to injury (53). M1-type macrophages are typically activated by IFNγ, either alone or in combination with LPS (54). Macrophages with the M1 phenotype highly express CD80, CD86, and CD16/32 (55), and are capable of producing pro-inflammatory cytokines such as IL-6, IL-12; chemokines such as CXCL9 and CXCL10; and NO (56). IL-4 and IL-13 induce the M2 macrophage phenotype, which is distinguished by its ability to produce vasoactive substances, exhibit anti-inflammatory properties, and promote tissue repair (57, 58). At the initial stage of HS formation, the phenotype of macrophages is predominantly of the M1 type. Under normal circumstances, the pro-inflammatory factors secreted by M1 macrophages function to clear pathogens and necrotic tissue in the initial wound environment. However, in HS, the overexpression of pro-inflammatory factors promotes the proliferation and differentiation of fibroblasts. Increasing evidence suggests that, during the early stages of wound healing, it is crucial to shift macrophages from the M1 pro-inflammatory phenotype to the M2 anti-inflammatory phenotype (59, 60).

Notably, the M1 and M2 classification model of macrophages is a simplified model for describing the different functional properties exhibited by macrophages during polarization. In addition, macrophages responding to the initial stimulus may exhibit a mixed phenotype between M1/M2, Mregs, and CXCL4-induced M4 type (53, 61, 62). However, these phenotypes have not been intensely studied in HS.

### 4.2 Medium-term trends and phenotypic shifts

As the inflammatory response develops or environmental factors change, the macrophage phenotype shifts in the medium term, a process that usually occurs within a few days to a week (63). At this point, macrophages can shift from M1 to M2 type or between M2 subtypes (**Figure 1F**). M2-type macrophages express specific surface markers, such as CD206 and CD204, and secrete anti-inflammatory factors, thereby inhibiting inflammation and promoting tissue regeneration and repair. M2 macrophages can be further categorized into M2a, M2b, M2c, and M2d subtypes (64, 65) (**Figure 1F**). Although current data suggest that different phenotypes of M2 macrophage subpopulations play different roles, no reports have evaluated the role of M2 macrophage subtypes in HS, and further studies are warranted.

Interestingly, one study suggests that tumors are somehow characteristic of unhealed wounds. Significant similarities exist between many tumor markers and the wound-healing process’s biological markers (66). In the tumor microenvironment, macrophages typically exhibit specific phenotypes associated with tumor progression. Tumor-associated macrophages (TAMs), which are highly plastic, can adopt either an M1-like phenotype, contributing to anti-tumor immunity, or an M2-like phenotype, promoting tumor growth and immune escape (67, 68). This mixture of phenotypes and their dynamic changes reflect the complex response of macrophages in different environments. Recent advances in cancer immunotherapy have demonstrated that targeting TAMs can effectively modulate their function. For instance, PD-1+ TAMs exhibit impaired phagocytic capacity, which can be rescued by PD-1/PD-L1 blockade, leading to a reduction in tumor burden (69, 70). Given the functional plasticity of macrophages in both tumor progression and wound healing, it is plausible that a similar immunomodulatory approach could be relevant to HS treatment. Future research should explore whether macrophages undergo a TAM-like phenotypic shift during HS formation and whether targeting immune checkpoints such as PD-1/PD-L1 could regulate macrophage activity to control excessive fibrosis and pathological scar formation. Investigating these mechanisms may provide novel insights into macrophage-targeted therapies for HS.

### 4.3 Late phenotype and persistence

Under prolonged inflammation lasting weeks to months, macrophages tend to adopt a phenotype suited to the specific tissue or pathological state, facilitating tissue stabilization and functional recovery. In this process, M2-type macrophages predominate (**Figure 1G**). By secreting anti-inflammatory cytokines, M2-type macrophages inhibit inflammatory responses and prevent tissue damage caused by excessive immune responses. In addition, M2-type macrophages secrete VEGF and PDGF during the recovery phase after injury, which promotes neovascularization and collagen production to support new tissue generation and repair. This M2-type phenotype tends to be persistent during the remodeling phase of HS formation. This results in excessive scar tissue formation via mechanisms such as prolonged anti-inflammatory response, fibrosis promotion, and ECM remodeling.

Besides, in long-standing chronic inflammatory or tumor environments, macrophages can also exhibit a mixture of M1 and M2 phenotypes (71) (**Figure 1G**). This mixed phenotype can sustain the fight against pathogens while modulating the local immune microenvironment to avoid excessive damage to normal tissues. Overall, the phenotypic changes of macrophages during the formative stages of HS further reflect their complex roles in maintaining immune homeostasis and regulating pathological states. Modulation of macrophage phenotypic switching is expected to be a new strategy for treating disease.

## 5 Macrophages affect HS through different pathways

### 5.1 Effect of M1-type macrophage-associated signaling molecules on HS

#### 5.1.1 TNF-α

**Figure 2:**
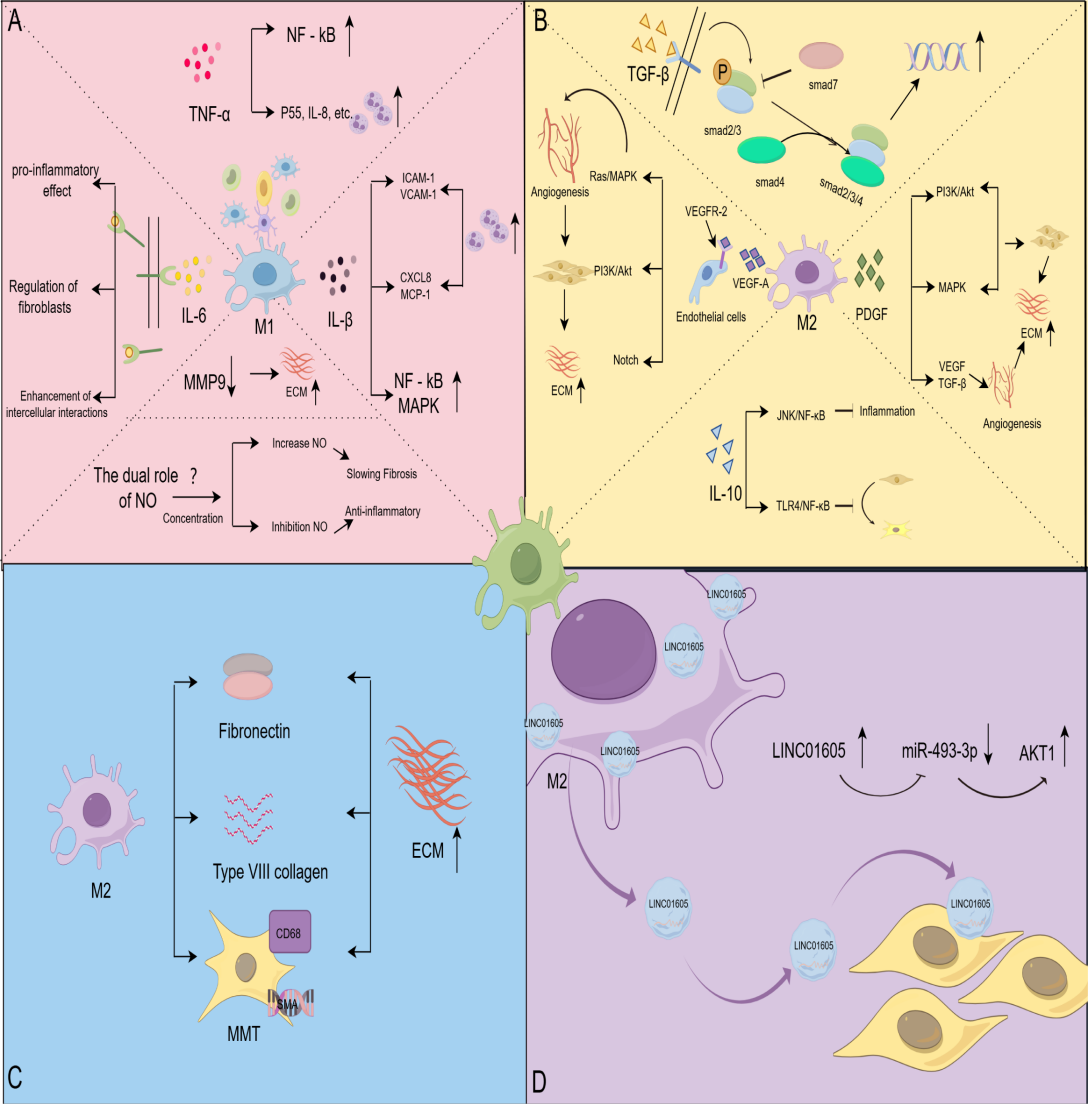
Macrophages affect HS through different pathways. **(A)** Effect of M1-type macrophage-associated signaling molecules on HS; TNF-α activates NF-κB and promotes inflammatory cell infiltration; IL-6 exhibits pro-inflammatory activity, regulates fibroblast behavior, and interacts with macrophages, fibroblasts, and endothelial cells; IL-1β recruits immune cells and activates the NF-κB and MAPK signaling pathways; NO regulates fibrotic disease in a concentration-dependent manner; MMP9 reduces ECM deposition. **(B)** Effect of M2-type macrophage-associated signaling molecules on HS; over-activation of the TGF-β pathway promotes fibroblast value-addition and differentiation, and Smad7 negatively regulates TGF-β signaling; IL-10 inhibits inflammatory responses and fibroblast differentiation through the JNK/NF-κB and TLR4/NF-κB pathways; VEGF activation Ras/MAPK, PI3K/Akt and Notch signaling pathways, which regulate the behavior of vascular endothelial cells and promote angiogenesis overgrowth; PDGFPI3K/Akt, MAPK and other signaling pathways enhance the secretory activity of fibroblasts, and synergistically act with VEGF and TGF-β to promote angiogenesis and promote the development of HS. **(C)** Macrophages were directly involved in matrix remodeling; macrophages secreted fibronectin and collagen type VIII and differentiated into myofibroblasts to directly intervene in ECM remodeling. **(D)** Macrophages mediate HS via exosome formation; exosomes derived from M2 macrophages were enriched in LINC01605, and high levels of LINC01605 caused a decrease in miR-493-3p and activated AKT to enhance the inflammatory response. (By Figdraw).

TNF-α is a pleiotropic cytokine secreted by various immune cells, including monocytes, T cells, dendritic cells, natural killer cells, and macrophages. M1-type macrophages are one of the significant sources of TNF-α (72). TNF-α regulates inflammatory responses and plays a crucial role in biological processes such as apoptosis, cell proliferation, and autophagy (73–75). A 16S rRNA sequencing showed that the expression of TNF-α was significantly higher in HS tissues than in normal tissues (76). TNF-α is involved in HS through the activation of relevant inflammatory pathways. The NF-kB pathway was shown to be activated in HS fibroblasts (77). Studies have shown that TNF-α induces ROS production in human dermal fibroblasts and upregulates the transcription factor NF-κB (78). *In vitro* experiments demonstrated that TNF-α induced up-regulation of MMP-1 and MMP-3 expression in human dermal fibroblasts. Resveratrol significantly inhibited this effect through the NF-κB pathway (79). Another study showed that blocking TNF-α interaction with TNFR effectively inhibits TNF-α-induced NF-κB activation (80). These studies suggest that TNF-α activates the NF-κB signaling pathway by TNFR, which alters the biological behavior of human dermal fibroblasts and may play a role in HS formation (**Figure 2A**).

In addition, TNF-α interacts with neutrophils to promote HS formation (**Figure 2A**). Research suggests that TNF-α activates neutrophils via IL-8, p55, secretory vesicles, and specific granules, enhancing their bactericidal activity and releasing inflammatory mediators (81, 82). In the early stages of infection and inflammation, TNF-α regulates the activity of neutrophil surface receptors, inhibits apoptosis, and prolongs the lifespan of neutrophils through signaling pathways such as JNK and NF-κB (83, 84). Notably, TNFR1 and TNFR2 initiate pro-inflammatory signaling, but only TNFR1 triggers a pro-apoptotic response (85). Moreover, TNF exhibits dual properties that activate neutrophil apoptosis under specific conditions (86, 87).

Although the mechanism of TNF-α has been extensively studied, TNF-α inhibitors are widely used in treating psoriasis, rheumatoid arthritis, and other immune-related diseases (88, 89). However, their specific role in HS lacks support from large-scale research data. Understanding the interactions between TNF-α and other inflammatory factors and cell types could help clarify its overall role in HS formation and potentially optimize treatment strategies.

#### 5.1.2 IL-6

IL-6 is a multifunctional cytokine that affects the fibrotic process by promoting monocyte recruitment, M2-type macrophage polarization, and increased ECM deposition (90). After binding to its receptor, IL-6 exerts its biological effects primarily through three signaling modes: cis, trans, and cluster signaling (91). Studies indicate that IL-6 is one of the most significant cytokines promoting human HS formation, highlighting its critical role in fibrotic pathology (25). Trans signaling by IL-6 exhibits pro-inflammatory activity and is associated with various pathological changes (92). Previous studies have demonstrated that activation of trans IL-6 signaling accelerates fibrosis (93). Inhibition of IL-6 and its downstream pathways positively impacts the clinical management of fibrotic diseases (94, 95). Interestingly, a study of renal scarring found no significant differences in trans IL-6 signaling markers between patients with renal scarring and those without scarring (96). This suggests that there may be some degree of dysfunction in the trans-IL-6 signaling pathway during scarring that deserves further investigation.

IL-6 regulates fibroblast behavior through autocrine mechanisms. *In vitro* experiments demonstrated that IL-6 autocrine activity drove scar fibroblasts to exhibit significant anisotropy and altered ECM arrangement, resulting in a directional matrix structure (97). This change in matrix structure and orientation may contribute to the rigid and irregular morphology of scar tissue. This study suggests that IL-6 not only promotes HS formation via its pro-inflammatory effects but also influences ECM structure by modulating fibroblast behavior. This highlights the importance of IL-6 signaling in inhibiting fibrosis progression as a potential therapeutic target.

Additionally, elevated IL-6 levels promote HS formation through intercellular interactions. Specifically, IL-6 stimulates macrophage polarization towards the M2 type, upregulates TGF-β and promotes fibroblast differentiation towards myofibroblasts (98). Furthermore, IL-6 amplifies the inflammatory response in vascular endothelial cells, regulating angiogenesis and immune cell recruitment. These effects are mediated by classical and trans signals activating PI3K-Akt, ERK1/2, and gp130 signaling pathways (99, 100). In summary, IL-6 plays a key role in HS formation through multiple signaling pathways and intercellular interactions (**Figure 2A**). However, the complexity of the IL-6 signaling pathway has not been fully clarified. Exploring the causes of dysregulation of the IL-6 signaling pathway and its specific role in the fibrosis process will help develop more precise therapeutic strategies.

#### 5.1.3 IL-1β

IL-1β is also highly expressed in HS (27). IL-1β belongs to the IL-1 family, which consists of 11 cytokines.IL-1β is one of these ligands with proinflammatory activity and is produced mainly by macrophages. In the early stages of scar formation, IL-1β attracts immune cells to migrate to the damaged site by activating the inflammatory response. It has been shown that IL-1β enhances vascular permeability and promotes the aggregation of inflammatory cells to the area of injury by upregulating the expression of ICAM-1 and VCAM-1, adhesion molecules of vascular endothelial cells (101, 102). IL-1β also stimulates endothelial cells to secrete CXCL8 and monocyte chemotactic protein-1, which further attracts immune cells, such as neutrophils and monocytes, to the site of infection or injury (103). In addition, IL-1β activates the NF-κB and MAPK signaling pathways, amplifying the inflammatory response and exacerbating tissue fibrosis (104, 105). Studies have shown that anti-IL-1β therapy is effective in inhibiting the course of chronic progressive fibrosis. This effect may be attributed to its inhibition of IL-1β-mediated pro-inflammatory responses (106, 107).

Overall, IL-1β exacerbates tissue fibrosis by enhancing the inflammatory response and attracting immune cell aggregation (**Figure 2A**). Although some preliminary studies have suggested a possible role for anti-IL-1β therapy in organ fibrotic diseases (107, 108), more clinical studies and trials are needed to determine the efficacy and applicability of anti-IL-1β therapy.

#### 5.1.4 iNOS

Endogenous nitric oxide (NO) is produced by three different types of enzymes: neuronal NOS (nNOS; NOS1), inducible NOS (iNOS; NOS2), and endothelial NOS (eNOS; NOS3) (109). Of these, iNOS plays a critical role in the fibrotic process. iNOS activity is upregulated by cytokines, such as IFN-γ and LPS, secreted by M1-type macrophages. This leads to the production of large amounts of NO, which is involved in regulating fibrosis and the inflammatory response. Recent studies have found that NO exhibits different effects in various fibrotic diseases through different concentrations and mechanisms (110) (**Figure 2A**). NO inhibition of myofibroblast activation and collagen I production in renal fibrosis, using nanocarrier-delivered NO, slows the progression of renal fibrosis (111). In a phase 2 clinical trial, pulsed inhaled NO demonstrated favorable safety and tolerability in treating interstitial lung disease and improved patients’ physical activity (112). These studies suggest the potential benefit of NO supplementation in fibrotic diseases.

In contrast, other studies have demonstrated that inhibiting NO production in LPS-stimulated RAW 264.7 macrophages exerts an anti-inflammatory effect (113). Huseyin Gungor’s study supports the notion that inhibiting NO production helps improve liver fibrosis (114). Another *in vitro* study showed that p53 knockdown mesenchymal stem cells (MSCs) promoted fibroblast proliferation by increasing NO production. This phenomenon was reversed by inhibiting NO production (115). It is thus clear that the role of NO in fibrotic diseases is dual, and its specific effect depends mainly on the concentration level of NO. Further research on the application of NO in fibrotic diseases is of critical significance for guiding HS treatment.

#### 5.1.5 MMPs

MMPs are a class of metal ion-dependent proteases capable of degrading a wide range of components in the ECM. The human genome contains 24 MMP genes, two of which encode the MMP23 protein, resulting in 23 distinct MMPs. Under normal conditions, MMP activity is low, but it increases significantly during tissue repair and inflammation. MMPs regulate tissue degradation and remodeling by cleaving ECM components such as collagen, fibronectin, laminin, and gelatin (116). Among MMPs, MMP-9 plays a crucial role in HS formation (**Figure 2A**). Previous studies have found that upregulated gene expression of MMP-2, MMP-9, and TIMP-1 is strongly associated with proliferative scarring (117). In experiments with a rabbit ear scar model, elevated MMP-2 and MMP-9 expression significantly reduced the Scar Elevation Index, Epidermal Thickness Index, and collagen deposition (118). Recent *in vitro* studies have demonstrated that capacitive resistive electro transfer therapy alters MMP-9 expression in human myofibroblast cultures, potentially benefiting fibrotic pathology treatment (119). These findings suggest that the activity of MMP-9 has a critical role in tissue repair. The mechanism of macrophage-MMP-ECM interactions warrants further investigation. Targeted interventions against this interaction may offer new strategies for treating HS.

### 5.2 Effect of M2-type macrophage-associated signaling molecules on HS

#### 5.2.1 TGF-β

TGF possesses the ability to induce a transformed phenotype in untransformed cells, and it was first discovered by De Larco and Todaro in 1978. Mammalian cells express three isoforms of TGF: TGF-β1, TGF-β2, and TGF-β3. These isoforms are widely involved in biological processes, such as inflammation, matrix generation, matrix remodeling, cell proliferation, and the regulation of apoptosis, all of which play an important regulatory role in HS formation (120, 121).

Recent studies have confirmed that TGF-β, especially the TGF-β1 isoform, significantly promotes HS formation. Specifically, TGF-β1 promotes fibroblast activation by activating the downstream Smad protein signaling pathway (**Figure 2A**). Simultaneously, it induces fibroblasts to secrete large amounts of collagen and fibronectin, which leads to the excessive accumulation of scar tissue (122). Studies have shown that inhibition of the activation of the TGF-β1-Smad2/3/4 signaling pathway promotes apoptosis of fibroblasts, thereby alleviating HS production (123). In addition, TGF-β1 promotes the differentiation of fibroblasts into myofibroblasts by regulating the expression of α-SMA, further enhancing contractility and fibrosis at the trauma site (124).

Several studies have shown that moderate TGF-β signaling during the early stages of wound repair helps maintain the tissue repair balance and promotes wound healing. In contrast, when TGF-β signaling is overactivated, it leads to fibrosis formation. Smad7 is an inhibitory factor in the TGF-β signaling pathway and plays a key role in the negative regulation of fibrosis. It inhibits the excessive transmission of TGF-β signaling by competitively inhibiting the phosphorylation and nuclear translocation of Smad2/3, thereby limiting the progression of fibrosis (125) (**Figure 2B**). These findings highlight the complex role of TGF-β signaling in wound repair, including its positive role in promoting wound healing but also the risk of fibrosis. Previous studies have shown that the critical role of TGF-β in scar formation has been extensively researched, and that TGF-β inhibitors have demonstrated potential efficacy in preclinical studies (126). However, since the TGF-β signaling pathway is critical for various normal physiological processes, such as wound healing and immune regulation, treatments directly targeting TGF-β may lead to significant side effects. Therefore, future studies may need to develop more selective TGF-β inhibitory strategies to minimize side effects while preserving therapeutic efficacy.

#### 5.2.2 IL-10

IL-10 is an anti-inflammatory cytokine, mainly secreted by M2-type macrophages, and it is involved in scar formation and fibrosis-related diseases. However, the anti-fibrotic molecular mechanism of IL-10 in skin scarring remains unclear. Early studies found that a lack of IL-10 in fetal skin triggered scar formation. This intrinsic lack of IL-10 may lead to a sustained amplification of inflammatory cytokines, persistent stimulation of fibroblasts, and abnormal collagen deposition (127). Other studies have demonstrated that IL-10 is highly expressed in fetal skin during mid-gestation and is absent in human skin after birth (128). These findings tentatively suggest that IL-10 is involved in wound healing and scar formation. Recently, several studies have shown that IL-10-modified BMSCs inhibited inflammatory progression through the JNK/NF-κB pathway and prevented the formation of HS in a rabbit ear model (129). This suggests that IL-10 may mediate the JNK/NF-κB pathway to exert an anti-scarring effect. The study by Xie Fang et al. demonstrated that IL-10-modified Adipose-Derived Mesenchymal Stem Cells prevented HS formation by modulating fibroblast biology and inflammation (130). Another *in vitro* study demonstrated that IL-10 regulates the TLR4/NF-κB pathway in dermal fibroblasts via the IL-10R/STAT3 axis, which in turn reduced ECM deposition and fibroblast-to-myofibroblast transformation, thereby attenuating LPS-induced HS formation (31).

In summary, IL-10 may take on an anti-scarring role through various mechanisms, such as reducing the inflammatory response and modulating the biological behavior of fibroblasts (**Figure 2B**). Although IL-10 has the potential to inhibit HS formation, it is poorly stable *in vivo* and requires an effective delivery system to ensure adequate concentration in target tissues. Existing delivery systems (e.g., nanocarriers, hydrogels, and other methods) are effective, but their effectiveness and safety must be further validated. Future research may focus on developing controlled-release IL-10 systems that can be combined with antifibrotic drugs, laser therapy, or other cytokines to develop a combination therapy strategy for preventing HS.

#### 5.2.3 VEGF

The process of HS formation is closely related to dysregulated angiogenesis. VEGF is the major pro-angiogenic factor, generating different mRNA variants through alternative splicing. These variants are translated to produce protein subtypes of different lengths and biological functions (131). Specifically, VEGF-A binds to VEGFR-2, forming a dimer and activating the receptor’s tyrosine kinase activity. This process triggers the autophosphorylation of tyrosine residues on the receptor. The phosphorylated tyrosine residues become binding sites for various downstream signaling molecules, activating multiple signaling pathways such as Ras/MAPK, PI3K/Akt, and Notch (132, 133) (**Figure 2B**). These pathways act synergistically to regulate the behavior of vascular endothelial cells and ultimately promote angiogenesis. However, overactivation of these signaling pathways can lead to excessive angiogenesis. The overproduced blood vessels provide sufficient nutritional support for the abnormal proliferation of fibroblasts and collagen deposition, thus exacerbating scarring. Numerous studies have shown that downregulation of VEGF signaling can alleviate HS formation (134, 135). Moreover, VEGF signaling also upregulates the expression of MMPs, promoting endothelial cell migration in the stroma and neovascularization (136, 137). Thus, VEGF and its associated signaling pathways play a multifaceted role in the formation of HS, making it an essential target for understanding and treating pathological scarring.

#### 5.2.4 PDGF

The PDGF family consists of PDGF-aa, PDGF-bb, PDGF-ab, PDGF-cc, and PDGF-dd, which are composed of five members that form disulfide-linked homo- or heterodimers (138). The primary sources of PDGF are platelets and fibroblasts, and M2 macrophages can secrete small amounts of PDGF during the proliferation phase of wound healing. PDGF regulates cell proliferation, migration, and differentiation by initiating downstream signaling pathways through binding to its specific receptor, PDGFR (139).

PDGF’s ability to promote fibroblast activity plays a crucial role in HS formation. Studies have shown that PDGF expression in HS tissue is significantly higher than in normal skin, which correlates directly with the hyperactivation of fibroblasts (140). The overexpression of PDGF leads to abnormal proliferation and migration of fibroblasts, resulting in collagen production exceeding normal levels and causing thickened and hardened scar tissue. Another *in vitro* study found that adding PDGF-BB to fibroblasts cultured *in vitro* significantly increased the proliferation rate of the cells (141). This effect was more pronounced in HS fibroblasts (142), suggesting an essential role of PDGF in promoting cell proliferation in this process.

PDGF also enhances the secretory activity of fibroblasts by activating the PI3K/Akt, MAPK, and other signaling pathways (**Figure 2B**). This results in the overproduction of collagen, fibronectin, and other ECM components, ultimately leading to the persistent and abnormal proliferation of scar tissue (143, 144). In addition, PDGF promotes HS generation through synergistic effects with VEGF and TGF-β (145, 146) (**Figure 2B**). This suggests that PDGF affects scar formation by directly acting on fibroblasts and also indirectly contributes to the development of HS by regulating angiogenesis. Inhibiting the PDGF/PDGFR signaling pathway can effectively block the aberrant signal transduction of various growth factors, thereby preventing the onset and progression of diseases such as fibrosis (147). Given the critical role of PDGF in HS, targeting the PDGF/PDGFR pathway is a promising therapeutic strategy.

### 5.3 Direct involvement of macrophages in matrix remodeling

Macrophages are not only indirectly involved in HS formation by secreting signaling molecules but also directly influence HS formation by remodeling the ECM. Macrophages directly intervene in ECM remodeling by both secreting collagen and differentiating into myofibroblasts (**Figure 2C**). As early as 1999, Weitkamp et al. used fluorescence techniques to demonstrate that macrophages can synthesize type VIII collagen themselves (148). Another study found that macrophages secrete fibronectin and collagen type VIII to promote ECM formation and express almost all known collagen and collagen-related mRNAs (149). These findings suggest a direct role for macrophages in HS formation.

Notably, macrophages undergo macrophage-to-myofibroblast trans differentiation (MMT) in chronic inflammation and fibrotic pathological environments. A study collected human liver specimens at different stages of hepatic fibrosis and found MMT cells, which co-expressed macrophage (CD68) and myofibroblast (a-SMA) markers. Moreover, this result was validated in an animal model of liver fibrosis (150). In another study, researchers found that macrophages are involved in the formation of subretinal fibrosis through MMT changes (151). This suggests that MMT is involved in the progression of multiple fibrotic diseases. However, current research on direct collagen secretion by macrophages has primarily focused on organ fibrosis. Therefore, there is an urgent need to improve the understanding of macrophage-secreted ECM components in skin scarring.

### 5.4 Macrophages mediate HS formation via exosomes

Macrophages secrete different extracellular vesicles, including Exosomes, Microvesicles, and Apoptotic Bodies. Exosomes are important extracellular vesicles that contain various bioactive molecules such as microRNA, proteins, and lipids, which can regulate intercellular communication and influence the behavior of recipient cells.

Exosomes are secreted by prokaryotic and eukaryotic cells, with a diameter of about 30-150 nm, and are essential carriers of paracrine signaling (152). Macrophages initiate the process through membrane endocytosis, forming endosomes. Subsequently, the endosomes generate intraluminal vesicles in the cytoplasm, which transform into multivesicular bodies (MVBs). Finally, the MVBs fuse with the cell membrane, releasing exosomes (152, 153). A study co-culturing M2 macrophages with human dermal fibroblasts found that exosomes derived from M2 macrophages promoted the proliferation and migration of human dermal fibroblasts by delivering LINC01605 (154) (**Figure 2D**). Another study showed that M2 macrophage-derived exosomes were enriched in long-stranded noncoding RNA, specifically lncRNA-ASLNCS5088. This lncRNA can be efficiently transferred to fibroblasts, increasing α-SMA expression (155). Interestingly, recent studies have shown that macrophage filamentous pseudopods can produce filopodia tip vesicles. Such vesicles detach from the tips of the cell’s filamentous pseudopods and deliver many molecular signals to fibroblasts (156). These studies suggest that macrophage-derived exosomes play a role in HS formation by influencing fibroblast behavior.

Although studies of macrophage-mediated HS formation via exosomes have shown potential, their high cost and shortcomings in delivery efficiency and specificity have limited their application. In the future, new breakthroughs in therapeutic HS should be achieved by studying standardized production and optimizing delivery systems.

## 6 Treatment strategies for HS

### 6.1 Emerging technologies for HS treatment

#### 6.1.1 Photomedical therapy technology

Compared with traditional conservative treatments, such as local drug injections and physical pressure therapy, photoelectric technology offers non-invasiveness, high precision, a rapid onset of action, and shorter treatment durations. Common photoelectric therapy modalities used in clinical practice include laser therapy (ablative and non-ablative), microplasma radiofrequency technology, and photodynamic therapy (PDT).Recently, PDT has emerged as a promising non-surgical strategy for treating HS in both cellular studies and animal models. PDT primarily relies on the cytotoxic effects of photosensitizers to achieve its therapeutic action. When photosensitizers accumulate around proliferating fibroblasts, laser irradiation triggers the production of reactive oxygen species (ROS).ROS exert cytotoxic effects on fibroblasts, inducing apoptosis and ultimately leading to the necrosis of scar tissue (157, 158). However, the detailed mechanism underlying PDT’s scar-inhibitory effects remains unclear. In clinical applications, PDT is often combined with microneedling techniques, which enhance drug penetration while amplifying PDT’s anti-scarring effects (159, 160). There is no doubt that PDT is an effective strategy for the prevention and treatment of HS. Notably, the selection of photosensitizers, the presence of side effects such as pain during treatment and how to effectively combine photodynamic therapy with other therapies are current challenges.

#### 6.1.2 New drug delivery systems

##### 6.1.2.1 Controlled release materials

Controlled-release materials significantly enhance the continuity and efficacy of therapy due to their unique drug slow-release properties. Hydrogel is a network structure composed of hydrophilic polymer chains linked by various chemical bonds and forces, featuring diverse cross-linking modes. In recent years, it has been used as a bioscaffold to promote wound healing, demonstrating promising therapeutic effects in the treatment of HS. Zivari-Ghader T et al. showed that hydrogel wound dressings made of chitosan/alginate scaffolds loaded with HPCE effectively prevented HS formation. This hydrogel exhibited antimicrobial, antioxidant, and anti-inflammatory properties, effectively inhibiting excessive collagen deposition and reducing inflammation (161). Similarly, numerous studies support these findings (162–164). Fu et al. demonstrated that hydrogels possess tension-shielding capabilities, which reduce wound tension via shape fixation and ultimately minimize HS formation (165). Zhang et al. utilized a bioglass/alginate composite hydrogel, which significantly inhibited scar formation in a rabbit ear scar model. The main mechanism involves stimulating the expression of the integrin subunit Alpha 2 in dermal fibroblasts, which accelerates wound healing and modulates fibroblast behavior (166). This indicates that different functional hydrogels can inhibit HS formation through multiple mechanisms (**Table 1**).

**Table 1:** Novel drug delivery systems for HS.

Material Type	Category	Name	Model	Mechanism	References
Controlled Release Materials	Hydrogel	Chitosan-Alginate Hydrogel with Hypericum perforatum Callus Extract	Mouse wound healing model/Normal human fibroblast cell	Inhibited E. coli and K. pneumoniae, MRSA, and MR-CoNS/accelerated re-epithelialization, neovascularization, and collagen deposition while reducing inflammation	(161)
		GelMA/PEGDA Hydrogel Microneedle Patch	Rabbit Ear HS Model/HS fibroblasts	Inhibition of HS fibroblasts/decreased the protein expression of collagen I/III and TGF-β1	(162)
		polysaccharide hydrogel	Rabbit Ear HS Model/Human keloid fibroblasts	Reducing the expression of α-SMA expression	(163)
		Tough, antibacterial, and antioxidant hydrogel	MRSA-infected rat full skin defect model/MRSA-infected rabbit ear HS model	Decreased inflammatory reactions, reduce collagen deposition, regulate collagen type and down-regulate α-SMA production	(164)
		Shape-fixing hydrogel	Mouse HS model	Reduces mechanical tension on wounds, optimizing the healing environment to promote scarless repair	(165)
		Bioglass/alginate composite hydrogels	Rabbit Ear HS Model/HS fibroblasts	Inducing scar fibroblasts apoptosis	(166)
	Microspheres	Porous microspheres loaded with asiaticoside	Epithelial cells, Dermal fibroblast cell models/Rat full-skin excision model	Accelerating re-epithelization, regulating the synthesis and disposition of different types of collagens	(167)
		Cellulose nanocrystal/calcium alginate-based porous microspheres	Mouse full thickness skin wound	Inhibited the activities of Escherichia coli, Staphylococcus aureus, and Pseudomonas aeruginosa	(168)
	Microsponges	Silver sulfadiazine-loaded microsponge gel	Epidermal keratinocyte and mouse embryonic fibroblast cell/Second degree burn wound model in mice	Enhanced the efficacy of the drug by reducing the cytotoxicity towards the keratinocytes and fibroblasts without altering the antimicrobial properties	(169)
		Resveratrol-loaded microsponge gel	Excision wound model in rats	Influenced cell adhesion	(170)
Enhanced Penetration Materials	Liposomes	anti-VEGF antibody-modified Paeonol liposome gels	Rabbit Ear HS Model	Inhibition inflammation	(171)
		Liposome-encapsulated statins	Rabbit Ear HS Model/Human foreskin fibroblasts	Decreased type I/III collagen content	(172)
	Ethosomes	ethosomes encapsulated with 5-florouracil	Rabbit Ear HS Model	CO2 fractional laser promote the permeation of 5-fluorouracil encapsulated ethosomes	(173)
		IR-808 loaded nanoethosomes	HS fibroblast/Rabbit Ear HS Model	promoting HSF apoptosis and remodeling collagen fibers	(174)
Bioactive Materials	Exosomes	LINC01605-enriched exosomes from M2 macrophages	Human dermal fibroblast	LINC01605 promoted fibrosis of human dermal fibroblast by directly inhibiting the secretion of miR-493-3p, and miR-493-3p down-regulated the expression of AKT1	(154)
		lncRNA-ASLNCS5088-enriched exosomes from M2 macrophages	Fibroblast	Inhibition fibroblast activation	(155)
		Exosome derived from mesenchymal stem cells	HS fibroblast	Inhibition the TNFSF-13/HSPG2 signaling pathway	(175)
		Exosomes from miR-29a-modified adipose-derived mesenchymal stem cells	Mouse scalded skin model/HS fibroblasts	Inhibition the TGF-β2/Smad3 signaling pathway	(176)
		Exosome from adipose-derived mesenchymal stem cells	Mice skin incision model/Fibroblasts model	Regulation of microRNA-181a/SIRT1 axis	(177)
		Exosomes from hypertrophic scar fibroblasts	Normal human keratinocytes	Changed molecular patterns of proliferation, activation, differentiation and apoptosis of NHKs and proliferation/differentiation regulators and EMT markers	(178)
		Exosomes derived from human hypertrophic scar fibroblasts	HS fibroblasts	Increased cell proliferation and migration,induces smad and TAK1 signaling	(179)
	Nanoparticles	Verteporfin-loaded bioadhesive nanoparticles	HS fibroblasts	Inhibition the collagen deposition and angiogenesis	(180)
		Resveratrol-laden mesoporous silica nanoparticles	HS fibroblasts	Induce the apoptosis and autosis via the ROS -mediated p38-MAPK/HIF-1α/p53 signaling axis	(181)
		DNA-Fe nanoparticle	Rabbit Ear HS Model/Human fibroblast cells	Remodeling collagen fibers and promoting human fibroblast cells apoptosis	(182)
		Cu2Se@LYC (CL) composite	Rabbit Ear HS Model/HS fibroblasts	Induce the generation of reactive oxygen species and mitochondrial damage in hypertrophic scar fibroblasts	(183)
		Cuprous oxide nanoparticles	Rabbit Ear HS Model/HS fibroblasts	Inhibiting HSFs proliferation and inducing HSFs apoptosis	(184)
	Nanofiber Membranes	Palmatine-loaded poly(ϵ-caprolactone)/gelatin nanofibrous scaffolds	Rabbit Ear HS Model/L929 Fibroblasts	Facilitate the adhesion, spreading and proliferation of L929 fibroblasts	(185)
		ginsenoside Rg3-loaded electrospun PLGA fibrous membranes	Rabbit Ear HS Model	Decreased collagen I, VEGF expression	(186)
		Random composite nanofibers	Rat whole skin defect model	Promote re-epithelialization and angiogenesis and reduce excessive inflammation	(187)
		Electrospun Naringin-Loaded Fibers	Normal Human Dermal Fibroblasts/Hypertrophic Human Fibroblasts	Decreased Normal Human Dermal Fibroblasts TGF-β1, COL1A1, α-SMA	(188)
		Electrospun Fibers Loaded with Pirfenidone	HS fibroblasts	Modulates the gene expression of TGF-β1 and α-SMA	(189)

Microspheres are small spherical multiparticulate drug delivery systems with diameters ranging from 1 to 1,000 μm, capable of enhancing the bioavailability, stability, and efficacy of traditional drugs while ensuring good safety. Zhang et al. constructed asiaticoside microspheres to achieve efficient drug loading and sustained release, providing regenerative healing and anti-scarring effects (167). Another study prepared hemostatic porous microspheres, which demonstrated high fluid absorption capacity and excellent coagulation properties, accelerating wound healing and highlighting their potential in scar treatment (168).

Microsponges are porous structures composed of polymerized particles, typically ranging from 5 to 300 μm in diameter. The porous structure of microsponges enables controlled drug release. In a study investigating a microsponge gel of silver sulfadiazine for burn wound treatment, loading silver sulfadiazine into a microsponge incorporated in a gel matrix enhanced drug potency, enabled sustained drug release, reduced dosing frequency, improved adherence in burn patients, and minimized cytotoxicity (169). Furthermore, incorporating microsponges into a hydrogel allows for sustained drug delivery to the wound site, while the gel matrix maintains a moist environment and enhances cell adhesion, promoting wound healing and offering a novel approach for HS treatment (170).

##### 6.1.2.2 Enhanced penetration materials

The stratum corneum is the primary barrier to drug delivery through the skin to the scar tissue. Both liposomes and ethosomes possess bilayer membrane structures that resemble biological membranes. This structure enables them to mimic the properties of biological membranes, facilitating their fusion with cell membranes, penetration of the stratum corneum, and enhancement of drug permeation. SHI et al. developed an anti-VEGF antibody-modified liposome gel containing salvinorin. It exhibited excellent skin permeability, delayed drug release, and promoted high drug accumulation in the dermis. *In vivo* studies demonstrated that it reduced VEGF, TGF-β1, and TNF-α levels, inhibited cell proliferation, and exhibited therapeutic effects on HS in a rabbit ear model (171). Xie et al. developed new statin-loaded liposomes with enhanced skin penetration, which were successfully delivered topically and significantly reduced HS formation in a rabbit ear model (172). Zhang et al. prepared 5-FU-encapsulated ethosomes for HS treatment in combination with CO2 fractional laser therapy. The nanoscale ethosomes penetrated scar tissue through narrow and tightly connected cellular gaps. Fractional laser reduces the required drug dose, facilitating drug penetration into deeper skin layers, achieving higher local concentrations, and effectively inhibiting HS formation (173). Similarly, Yu et al. utilized the transdermal delivery capability of ethosomes to prepare an IR-808-loaded nanoethosome system as a novel photosensitizer for HS treatment with transdermal photodynamic therapy (174).

##### 6.1.2.3 Bioactive materials

The exceptional biocompatibility and functionality of bioactive materials facilitate the effective repair of scar tissue. Exosomes, as critical mediators of intercellular communication, can deliver biologically active substances and have demonstrated significant therapeutic potential in the treatment of HS. Recent research on adipose stem cell exosomes (ADSC-exos) has yielded increasing evidence that ADSC-exos not only promote wound repair but also possess therapeutic potential for HS. By carrying specific microRNAs, ADSC-exos regulate target gene expression, suppress fibrosis-related signaling pathways such as TGF-β/Smad, reduce fibroblast proliferation, migration, and collagen deposition, and promote HS tissue repair. This demonstrates the great potential of ADSC-exos in the treatment of HS (175–177). Moreover, fibroblast exosomes have shown a beneficial role in HS treatment. They affect HS formation by regulating fibrotic signaling pathways, promoting cell proliferation migration and epithelial-mesenchymal transition (178, 179). Notably, lncRNAs enriched in M2-type macrophage-derived exosomes were found to influence HS formation through a mechanism potentially linked to fibroblast activation (154, 155).

Bioactive nanomaterials exhibit tremendous potential for treating proliferative scarring. Studies have utilized nanoparticles as encapsulants, effectively inhibiting scar tissue formation. For example, Rerteporfin, Resveratrol, and Doxorubicin hydrochloride were encapsulated into nanoparticles to enhance drug stability and targeting, reduce side effects, and inhibit scar fibroblast proliferation effectively (180–182). Additionally, nanoparticles have been used to deliver photosensitizers in combination with near-infrared light therapy to induce mitochondrial damage and cell death (183). Furthermore, nanoparticles have been found to regulate the proliferation and apoptosis of HS fibroblasts, providing a scientific basis for developing novel therapeutic strategies for HS (184). Beyond nanoparticles, nanofiber membranes play a critical role in HS treatment. One study prepared Palmatine-loaded electrospun poly(ϵ-caprolactone)/gelatin nanofibrous scaffolds. These scaffolds exhibited strong antimicrobial and antioxidant activities, significantly inhibited scar formation, and accelerated wound healing (185). Another study developed ginsenoside Rg3-loaded electrospun PLGA fibrous membranes using electrostatic spinning and pressure-driven infiltration techniques. These membranes promoted tissue repair during the early stage of wound healing and inhibited scar formation during the later stage (186). In addition, nanofibrous membranes can influence scar formation by regulating macrophage function and promoting macrophage polarization. In a study, dendritic mesoporous bioglass nanoparticles loaded with VR23 were blended with poly (ester-curcumin-urethane) urea to prepare random composite nanofibers with bi-directional modulation. The dressing effectively promoted scarless healing of chronic wounds (187).

In addition to serving as drug carriers, the structural and mechanical properties of nanofiber membranes play a crucial role in scar treatment. One study developed electrospun fibers loaded with naringin. The fibers featured an innovative rounded texture, which effectively minimized HS formation during early wound healing (188). Another biofiber loaded with Pirfenidone exhibited outstanding elongation and toughness, enabling it to effectively treat HS during the established wound healing phase (189).

Clinical treatment of HS has made significant progress fueled by advancements in optoelectronics and novel drug delivery systems, but these emerging technologies still face multiple challenges in practical application. With its non-invasive and minimally invasive characteristics, photofacial technology provides an innovative and promising approach to treating scarring. However, the technical complexity, high equipment costs, and stringent requirements for operator expertise have restricted its widespread adoption. Moreover, the efficacy of photoelectric treatment is often unpredictable due to patient-specific variability, and its long-term effects require further validation. Therefore, reducing treatment costs, improving operational simplicity, and ensuring treatment efficacy have become pressing challenges for the application of photoelectric technology in HS therapy.

On the other hand, novel drug delivery systems offer a more targeted and efficient method of administering drugs for HS therapy. However, the complex preparation processes of these novel delivery systems impose higher requirements on the biocompatibility, targeting, and stability of the materials. Poor biocompatibility may trigger immune responses, while inadequate targeting can result in non-specific drug distribution in normal tissues, raising the risk of side effects. Additionally, the stability of novel delivery systems *in vivo* is a critical determinant of their therapeutic efficacy. Therefore, optimizing the preparation process and enhancing delivery efficiency while ensuring material safety and efficacy have become major challenges for novel drug delivery systems in HS therapy.

### 6.2 Strategies for targeting macrophages in the treatment of HS

**Figure 3:**
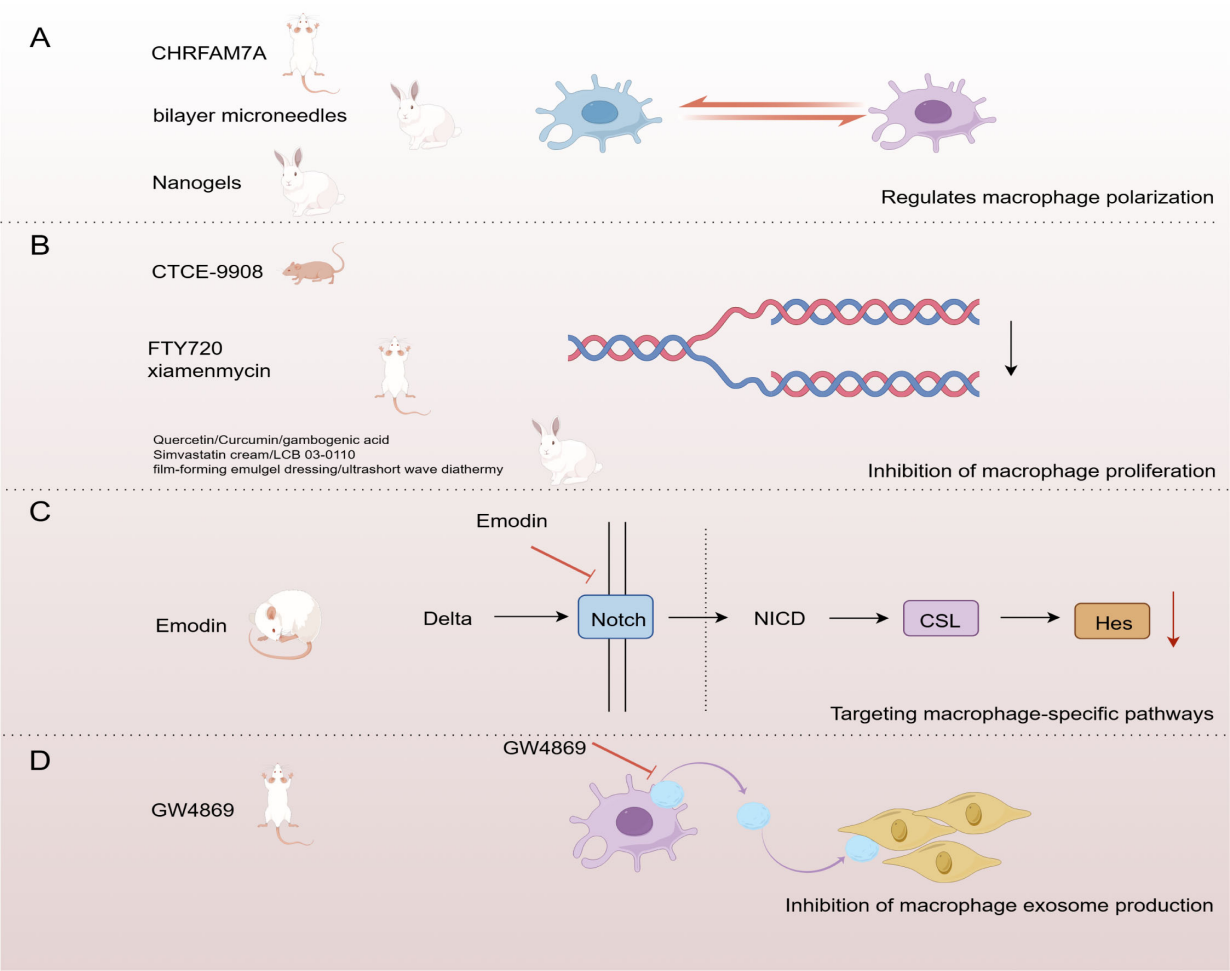
Strategies for targeting macrophages in the treatment of HS. **(A)** Regulation of macrophage polarization **(B)** Inhibition of macrophage proliferation **(C)**Targeting macrophage-specific pathways **(D)** Inhibition of macrophage exosome production. (By Figdraw).

Current treatment of HS focuses on controlling the inflammatory response with steroids and NSAIDs, as well as managing scar tissue through physical interventions such as surgery (190, 191). Although these methods are effective to a certain extent, they still have the limitations of significant side effects and high recurrence rates. Therefore, the development of new treatment modalities is urgently necessary. The role of macrophages in scar formation is gaining attention. Macrophages play a dual role in tissue repair, promoting both the inflammatory response and facilitating tissue remodeling. Therefore, therapeutic strategies targeting macrophages have emerged as a potential approach to mitigate proliferative scar formation. This review will discuss the strategies and prospects of targeting macrophages to treat proliferative scarring from four aspects (**Figure 3**, **Table 2**).

**Table 2:** Strategies for targeting macrophages in the treatment of HS.

Modes of action	Drugs/Methods	Model	Mechanisms of action	References
Regulation of macrophage polarization	CHRFAM7A	Mouse HS model of human skin grafts	Modulates macrophage phenotype and attenuates M2 macrophage activation	(194)
	Integrated bilayer microneedles	Rabbit Ear HS Model	Inhibition of macrophage M1 polarization	(193)
	Transdermal Transfersome Nanogels	Rabbit Ear HS Model	Promoting macrophage phenotype switching	(192)
Inhibition of macrophage proliferation	Film-forming emulgel dressing	Rabbit Ear HS Model	Decreased number of macrophages	(196)
	Quercetin	Rabbit Ear HS Model	Decreased number of macrophages	(197)
	Ultrashort wave diathermy	Rabbit Ear HS Model	Reduced macrophage ratio	(198)
	FTY720	Mechanical force induced HS model in mouse	Reduced M2-dominant macrophage frequency	(203)
	CTCE-9908	Nude mouse HS model of human xenografts	Decreased number of macrophages	(205)
	Simvastatin cream	Rabbit Ear HS Model	Reduced macrophage density	(201)
	Curcumin	Rabbit Ear HS Model	Decrease in the number of M2-type macrophages	(200)
	Gambogenic acid	Rabbit Ear HS Model	Reduced macrophage infiltration	(199)
	xiamenmycin	Mechanical force induced HS model in mouse	Decreased number of macrophages	(204)
	LCB 03-0110	Rabbit Ear HS Model	Decreased number of macrophages	(202)
Targeting macrophage-specific pathways	Emodin	Tail HS model in rats	Inhibition of macrophage recruitment and polarization/inhibition of Notch pathway	(207)
Inhibition of macrophage exosome production	GW4869	Mouse wound splint HS model	Blockade of lncRNA-ASLNCS5088-enriched exosome production in M2 macrophages	(155)

#### 6.2.1 Regulation of macrophage polarization

Dysregulation of the macrophage M1/M2 phenotypic transition is one of the major causes of HS formation. Thus, regulating the polarization state of macrophages is expected to inhibit fibroplasia and reduce scar formation. A study used nanogels as carriers to deliver tretinoin and 5-fluorouracil subcutaneously to a rabbit ear HS model. The results showed that this method effectively modulated macrophage phenotypic switching and had an antifibrotic effect, providing a promising therapeutic strategy for HS (192). Other studies have shown that a single dose of a two-layer microneedle system enhances the therapeutic effect against HS. The mechanism may be related to the anti-inflammatory drug dexamethasone, released from the outer layer, which inhibits the polarization of macrophages into a pro-inflammatory phenotype (193). Research by Tianya Li et al. also demonstrated that inhibiting the excessive polarization of M2 macrophages can effectively reduce scar formation (194) (**Figure 3A**). These findings suggest that therapeutic strategies targeting the modulation of macrophage polarization status have significant potential for preventing and treating HS. This provides new ideas for developing more effective anti-scarring therapies in the future.

#### 6.2.2 Inhibition of macrophage proliferation

Macrophage proliferation and accumulation have been noted as critical factors in the formation of HS (195). Therefore, inhibition of macrophage proliferation is considered a promising therapeutic strategy. Multiple studies have explored the effects of different drugs on macrophage proliferation and accumulation using a rabbit ear HS model. SFN/ASA-containing gel dressing and quercetin have been shown to inhibit scar formation by reducing macrophage numbers in a rabbit ear HS model (196, 197). In addition, ultrashort-wave hyperthermia reduced the macrophage ratio, while gambogenic acid decreased macrophage infiltration. Both demonstrated significant anti-scarring effects in this model (198, 199). Finally, simvastatin cream, curcumin, and LCB 03-0110 in a rabbit ear HS model also showed that reducing macrophage accumulation significantly inhibited fibrosis (200–202).

In a mechanical force-induced mouse model of HS, FTY720 significantly inhibited scar formation by reducing M2 -dominant macrophages (203). Xiamenmycin showed sound anti-scarring effects in this model by reducing macrophage retention (204) (**Figure 3B**).

Another study used a nude mouse model of HS generated by human xenografts and found that CTCE-9908 effectively controlled scar formation by reducing macrophage accumulation (205). These studies suggest that HS formation can be effectively controlled by targeting macrophage proliferation and accumulation, providing a new direction for future anti-scarring therapies.

#### 6.2.3 Targeting macrophage-specific pathways

Targeting macrophage-specific pathways is one of the critical strategies for treating HS. The Notch signaling pathway plays a role in dermal fibrosis by regulating fibroblast proliferation and activation, influencing inflammatory responses, and controlling ECM remodeling (206). It was shown that emodin significantly attenuated HS formation in the rat tail by inhibiting macrophage recruitment and polarization, an effect associated with inhibition of the Notch signaling pathway. This study revealed that down-regulation of Notch1, Notch4, and Hes1 could inhibit macrophage polarization and attenuate HS formation (207) (**Figure 3C**). This suggests that targeting the Notch pathway may be an effective intervention strategy for HS formation.

#### 6.2.4 Inhibition of macrophage exosome production

Therapeutic strategies to inhibit exosome production have shown potential in various diseases, including cancer, neurodegenerative diseases, and cardiovascular diseases (208, 209). GW4869 is a selective neutral sphingomyelinase inhibitor widely used to study the role of exosomes in disease by blocking their production (210). Studies have shown that GW4869 successfully inhibited the activation of fibroblasts by M2 macrophages by blocking the production of exosomes enriched with the long-chain lncRNA ASLNCS5088 from M2 macrophages. This mechanism was validated in an *in vitro* macrophage-fibroblast co-culture model and a mouse wound splint HS model, demonstrating its potential therapeutic value in inhibiting scar formation (155) (**Figure 3D**). These findings suggest that inhibition of exosome production may be a new direction for treating HS.

In summary, therapeutic strategies targeting macrophages demonstrate significant potential in managing HS. Modulating macrophage polarization status, inhibiting their proliferation, targeting specific signaling pathways, and inhibiting macrophage exosome production effectively reduce scar formation. Future studies should optimize strategies for targeting macrophages through further research and technological improvements to achieve more effective scar management in clinical practice.

## 7 Summary and outlook

HS formation is a complex pathological process involving the interaction of multiple cell types and signaling pathways. Macrophages play a key role at different stages as an important part of the innate immune system. This paper reviews the phenotypic changes of macrophages during HS formation, the effects of key signaling molecules on HS, and the potential of macrophages as therapeutic targets, revealing the importance of macrophages in forming and regulating scarring. However, there are still some questions about the role of macrophages in HS.

First, most studies have shown that M1-type macrophages promote the maintenance and expansion of the inflammatory response, while M2-type macrophages drive the formation and remodeling of scar tissue. However, the diverse roles of macrophages in HS are influenced by various factors, including the severity of the condition and variations in disease models. Currently, there is a lack of a standardized model for HS. Therefore, standardizing the methods for HS modeling is necessary to study the role of macrophages in HS better. Our review emphasizes the complex role of macrophages in HS formation, highlighting their dynamic phenotypic changes and their interactions with the scar microenvironment. These findings provide valuable guidance for the development of physiologically relevant and standardized HS models. By simulating the phenotypic changes of macrophages at different stages and their effects on the scar microenvironment, the physiological relevance of HS models and the translational value in preclinical research can be significantly enhanced. This not only facilitates the study of HS pathophysiological mechanisms but also provides new directions for the evaluation and optimization of emerging therapeutic strategies.

Second, this paper reviews the direct effects of macrophages on HS formation through various signaling molecules. However, macrophage-associated signaling molecules such as TNF-α, IL-6, TGF-β, and IL-10 may play dual roles in different pathological settings. Therefore, further studies on the mechanisms by which macrophage-associated signaling molecules function are necessary.

Third, macrophage-targeted therapies are considered a promising strategy for managing HS due to their critical role in scar formation and remodeling. By precisely regulating macrophage polarization and function, these therapies can reduce scar formation and improve wound healing outcomes. The integration of macrophage-targeted therapies into existing HS management practices has the potential to enhance treatment efficacy. For instance, combining macrophage-targeted therapies with laser therapy or novel delivery systems can provide more precise, localized, and sustained therapeutic effects while minimizing the systemic side effects commonly associated with traditional treatments. However, successful clinical application still requires addressing individual variability in patient responses, challenges related to cost-effectiveness and affordability, and rigorous clinical trials to validate their efficacy and safety. Overall, targeting macrophages is expected to be a new and effective therapeutic strategy for preventing HS.
